# Effect of the Duration of Restrictive Fluid Therapy on Acute Kidney Injury in Robot-Assisted Laparoscopic Prostatectomy

**DOI:** 10.3390/jpm13121666

**Published:** 2023-11-28

**Authors:** Serap Aktas Yildirim, Zeynep Tugce Sarikaya, Lerzan Dogan, Bulent Gucyetmez, Levent Turkeri, Fevzi Toraman

**Affiliations:** 1 Department of Anesthesiology and Reanimation, School of Medicine, Acibadem Mehmet Ali Aydinlar University, Istanbul 34752, Turkey; 2 Department of Anesthesiology and Reanimation, Acibadem Altunizade Hospital, Istanbul 34662, Turkey; 3 Department of Urology, School of Medicine, Acibadem Mehmet Ali Aydinlar University, Istanbul 34662, Turkey

**Keywords:** acute kidney injury, robot-assisted laparoscopic prostatectomy, restrictive fluid therapy, stroke volume index

## Abstract

Background: In robot-assisted laparoscopic prostatectomy (RALP), restrictive fluid therapy (RFT) is often utilized until the vesicourethral anastomosis (console period) is completed. RFT can cause acute kidney injury (AKI). Thus, RFT prolongation in surgeries that utilize the Trendelenburg position and pneumoperitoneum may increase the risk of postoperative AKI. We aimed to evaluate the effect of RFT duration on postoperative AKI. Methods: Forty-four patients who underwent RALP were included in this prospective observational study. Patients were divided into two groups according to the RFT duration (Group I, RFT duration ≤ 3 h, and Group II, RFT duration >3 h). AKI was diagnosed and staged according to the Kidney Disease Improving Global Outcomes criteria (KDIGO) using patients’ serum creatinine levels after the first 24 h postoperatively. Hemodynamic parameters were monitored using the pressure recording analytical method. Results: The AKI incidence was significantly higher in Group II than in Group I (45.5% vs. 9.1%; *p* = 0.016). In both groups, all patients who developed AKI were KDIGO stage 1 and all recovered on the second postoperative day. At the end of the console period, the heart rate and arterial elastance were significantly higher, whereas the stroke volume index was significantly lower in Group II than in Group I (*p* = 0.041, *p* = 0.016, and *p* < 0.001, respectively). Although the amounts of fluid administered before and after the anastomosis were similar between the groups, the total amount of fluid administered was significantly different (*p* < 0.001). There was a significant negative correlation between RFT duration and the total amount of fluid administered (r^2^ = 0.43, *p* < 0.001). RFT duration of >3 h, total fluid administration of ≤3.3 mL/kg/h, and stroke volume index (SVI) at the end of the console period of ≤32 mL/m^2^ increased the risk of AKI by 12.0 times (1.7–85.2) (*p* = 0.013). Conclusion: RFT prolongation in RALP may increase the risk of developing AKI.

## 1. Introduction

Robot-assisted laparoscopic prostatectomy (RALP) is the second most frequently performed robotic surgery worldwide and the “new” gold standard due to its minimal invasiveness and good short-term results [1,2]. Therefore, it is imperative that anesthesiologists are aware of the pathophysiological changes that may occur during and after RALP.

In patients undergoing RALP, pneumoperitoneum, the deep Trendelenburg position, and fluid restriction to reduce urine output until the vesicourethral anastomosis is performed, make managing anesthesia challenging [3]. Fluid restriction during the console period, in which the surgeon controls the arms of the operating cart using his/her hands and feet, and when performing urethrovesical anastomosis is important; as minimal as possible urine flow is required to keep the surgical area dry and visible [4].

The prolongation of the robotic console time also causes the prolongation of fluid restriction, pneumoperitoneum, and time in the Trendelenburg position. This combination may cause significant pathophysiological changes in both the renal and cardiovascular systems and may lead to postoperative acute kidney injury (AKI) [5]. Previous studies have shown conflicting results regarding the effect of intraoperative fluid restriction on renal outcomes in patients undergoing RALP; in addition, few previous studies have investigated the impact of the length of robotic console time and RFT duration on the risk of AKI [6,7].

In this study, we aimed to determine the effect of the RFT duration during RALP on the incidence of postoperative AKI by intraoperatively monitoring patients using the pressure recording analytical method (PRAM). Additionally, we examined intraoperative hemodynamic findings that could predict the risk of AKI in patients who underwent RALP and were administered RFT.

## 2. Materials and Methods

### 2.1. Ethics Approval

This prospective, single-center observational study was conducted between September 2023 and October 2023 at the Altunizade Acibadem Hospital. The study was approved by the Ethics Committee of the Acibadem MAA University (No: ATADEK-2021-01/29).

### 2.2. Trial Registration

The trial was prospectively registered (No: NCT06000098) on 15 September 2023.

### 2.3. Patients

A total of 44 patients with an ASA physical status of 1–3, who were older than 18 years of age, and who were scheduled to undergo RALP surgery for malignant prostate cancer at Altunizade Acibadem Hospital were included in the study. Informed consent was obtained from all participants. Patients aged < 18 years as well as patients with chronic renal failure, renal disease, and AKI were excluded from the study. During hemodynamic monitoring with the PRAM, an inappropriate signal acquisition may occur due to some diseases and conditions (e.g., severe lung and severe valvular heart diseases). Therefore, patients with severe lung disease, rhythm disorders (e.g., atrial fibrillation, frequent premature beats), or severe valvular heart disease were also excluded from the study. Additionally, patients with myocardial infarction in the last three months were excluded from the study due to the possible difficulties in performing RFT and the need for vasopressors and inotropes.

According to the RFT duration, the patients were divided into two groups: those with an RFT duration of ≤3 h and those with an RFT duration of >3 h.

### 2.4. Study Protocol

#### 2.4.1. Anesthesia

Patients were taken up to the operating room after restricting food intake for at least eight hours and water intake for two hours. No intravenous (IV) fluids were administered before the induction of anesthesia. All patients were administered premedication (0.03 mg/kg of midazolam), and the radial artery was cannulated using a 20 G catheter under local anesthesia. Hemodynamic changes were monitored using an uncalibrated pulse contour device (MostCare; Vytech, Vygon, Padova, Italy).

After anesthesia induction with IV boluses of 2 mg/kg propofol and 1 μg/kg remifentanil, the patients were intubated following the IV administration of 0.6 mg/kg rocuronium for neuromuscular blockade. Following orotracheal intubation, the respiratory frequency was adjusted so that the tidal volume was 6–8 mL/kg and the end-expiratory carbon dioxide percentage was 30–40 mmHg. Positive end-expiratory pressure was set to 5 cm H_2_O, and mechanical ventilation was initiated in the volume-controlled mode.

Anesthesia was maintained with an oxygen/air mixture, a 40% end-expiratory oxygen percentage, and sevoflurane inhalation at 0.9–1 minimum alveolar concentration. Remifentanil (0.02–0.5 μg/kg/min) and volatile anesthetics (sevoflurane) were used to sustain the general anesthesia. Anesthesia depth was monitored using the Bispectral Index (BIS monitor; Covidien Medical, Boulder, CO, USA), which was kept at 40–50.

At the end of the surgery, anesthesia was terminated and Sugammadex was administered (2 mg/kg). Patients who opened their eyes with verbal stimuli and demonstrated sufficient muscle strength and respiratory effort were extubated.

#### 2.4.2. Fluid Administration

As a standard, 1–1.5 mL/kg/hour of crystalloid fluid was administered to the patients until vesicourethral anastomosis was achieved. Thereafter, RFT was terminated, and a liberal fluid regimen (8–10 mL/kg/h) was initiated until the surgery was completed. After the surgery, crystalloid solution was maintained at 1.5 mL/kg/h until the 2nd postoperative day. A mean arterial pressure (MAP) of <65 mmHg was considered hypotension. If hypotension developed, the arterial blood pressure was maintained using norepinephrine, ephedrine without exceeding the maximum of 30% of its preinduction value.

#### 2.4.3. Surgery

An experienced urologist used the Da Vinci Xi surgical system (DaVinci; Intuitive Surgical, Sunnyvale, CA, USA) to perform RALP with or without pelvic node dissection. In all the patients, the bladder neck-sparing approach was utilized. Pneumoperitoneum was achieved by insufflating the abdomen with carbon dioxide (12 mmHg), with the patients in the supine position. Subsequently, the patients were placed in the Trendelenburg position (45° from the horizontal). The vesicourethral anastomosis was performed after the prostate gland was removed. Before skin closure, the carbon dioxide was released. The skin incision was closed while the patient was positioned supine.

#### 2.4.4. Hemodynamic Monitoring

Patients were monitored with the PRAM, using the Most Care^®^ monitoring system. The following four intraoperative time points were selected to record the patients’ hemodynamic data: before anesthesia induction, ten minutes after anesthesia induction, at the 1st hour in the Trendelenburg position, and at the end of the console period. The following data were recorded at each time point: heart rate (HR), systolic arterial pressure (SAP), MAP, diastolic arterial pressure (DAP), arterial elastance (Ea), stroke volume variation (SVV), pulse pressure variation (PPV), dp/dt, cardiac index (CI), cardiac power output (CPO), and cardiac cycle efficiency (CCE).

#### 2.4.5. Additional Recorded Parameters

The demographic data of the patients were recorded. The following perioperative data were collected: operative time, console time, vasopressor drug administration, diuretic administration, amount of fluid administered intraoperatively, fluid intake (IV and oral) in the first 72 h postoperatively, intraoperative adverse cardiac events (hypotension, arrhythmia, and ischemic changes on ECG), postoperative complications, and the length of hospital stay. The patient’s hemoglobin, and hematocrit levels were measured preoperatively and postoperatively. Additionally, the troponin-I levels were recorded at the 24th hour postoperatively.

In both groups, serum creatinine concentration was measured at three time points: preoperatively, at 24 h postoperatively, and at 48 h postoperatively. Postoperative AKI was diagnosed and staged based on patients’ serum creatinine levels according to the KDIGO criteria [8] using the follow-up data of the patients after the first 24 h. During the postoperative period, urine output could not be monitored effectively due to continuous bladder irrigation. Therefore, urine output could not be used as a diagnostic parameter for postoperative AKI.

### 2.5. Statistical Analysis

Descriptive data are presented as mean ± standard deviation, median (quartiles), and percentages. The Shapiro–Wilcox test was used to detect normal distribution of data. The student-t, Mann–Whitney U, and chi-square tests were used to compare the two groups. The Pearson correlation test was used to determine a correlation between the console period and total fluid administration. ROC curve analysis was used to determine the cut-off and area under the curve (AUC) values for AKI. The chi-square test was also used to determine the relative risks for AKI. The sample size for each group was determined to be 22 (exact test, the difference between postoperative 24th hour AKI percentages of groups was 30%; ∝ = 0.05; power = 0.80) using G-power (version 3.1.9.4; Heinrich Heine University, Düsseldorf, Germany). SPSS (version 29; SPSS Inc., Chicago, IL, USA) was used for all statistical analyses, and a *p*-value of <0.05 was considered statistically significant.

## 3. Results

In both groups, the demographic data, comorbidities, and preoperative laboratory test results were similar (Table 1). The baseline hemodynamic parameters, pre- and post-anastomosis fluid administration values, total fluid intake (oral and IV) in the first 72 h following surgery, usage of vasoactive drugs, and number of patients who developed intraoperative adverse cardiac events were also similar between the groups (Table 2).

The operative time, time in the Trendelenburg position, time on the console, and AKI incidence were significantly higher in patients who received >3 h of RFT than in those who received ≤3 h of RFT (*p* < 0.001, *p* < 0.001, *p* < 0.001, and *p* = 0.016, respectively). However, total fluid administered was significantly lower in patients who received >3 h of RFT than in those who received ≤3 h of RFT (*p* < 0.001) (Table 2). The incidence of AKI was 9.1% and 45.5% in patients who received ≤3 h and >3 h of RFT, respectively. In both groups, the patients who developed postoperative AKI were diagnosed with KDGO AKI stage 1 according to the serum creatinine value at 24 h, and all patients recovered on the postoperative second day.

The hemodynamic parameter values after anesthesia induction and at the first hour in the Trendelenburg position were similar in both groups. However, at the end of the console period, the HR and Ea were significantly higher in patients who received >3 h of RFT than in those who received ≤3 h of RFT (*p* = 0.041 and *p* = 0.016, respectively). However, the SVI was significantly lower in patients who received >3 h of RFT than in those who received ≤3 h of RFT (*p* < 0.001) (Table 3).

**Figure 1:**
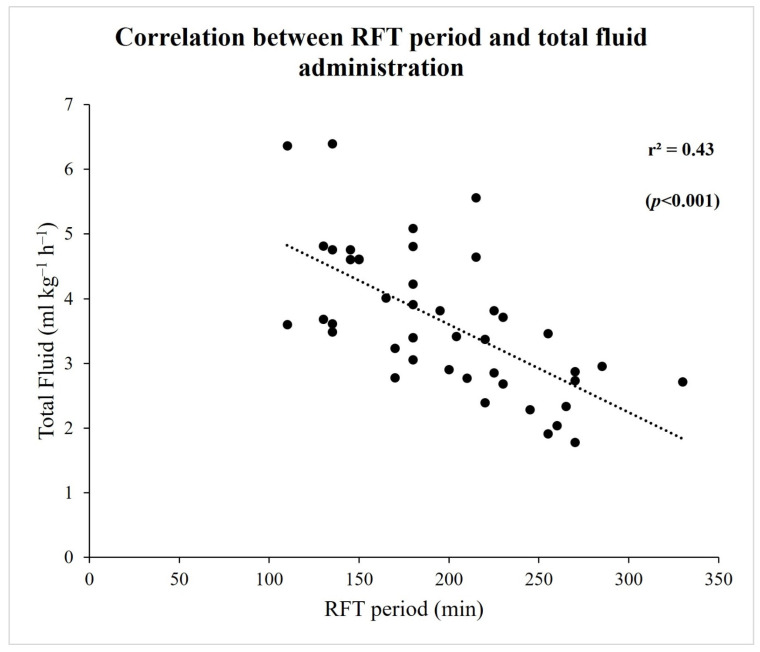
Correlation between RFT period and total fluid administration.

**Figure 2:**
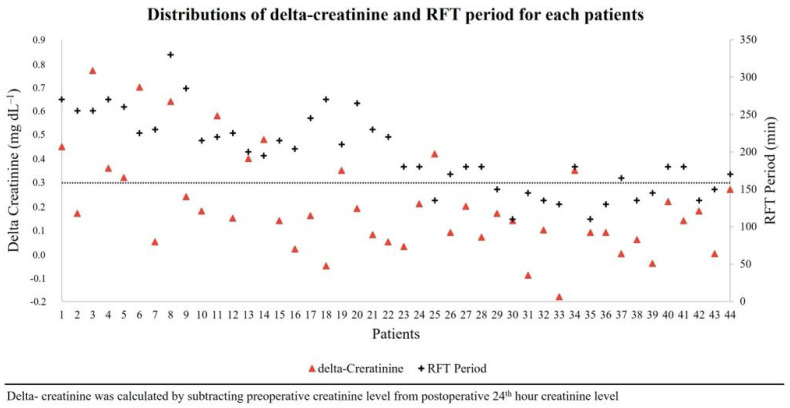
Distribution of delta-creatinine and RFT period for each patient.

There was a significantly negative correlation between the RFT duration and total fluid administered (r2 = 0.43, *p* < 0.001) (Figure 1). The distribution of delta-creatinine and RFT period for each patient is shown in Figure 2. For AKI in all patients, cut-off and AUC (CI 95%) values of total fluid administration and SVI at the end of the RFT period were ≤3.3 mL/kg/h (0.86 [0.74–0.98], *p* < 0.001) and ≤32 mL/m^2^ (0.72 [0.54–0.89], *p* = 0.029), respectively. There was no significant relative risk for AKI if only an RFT duration of >3 h was considered. However, the risk for AKI increased by 10.8-fold (1.4–82.6) if the RFT duration was >3 h and the total fluid administered was ≤3.3 mL/kg/h (*p* = 0.022). Additionally, the risk for AKI increased by 12.0-fold (1.7–85.2) if the RFT duration was >3 h, the total fluid administered was ≤3.3 mL/kg/h, and the SVI at the end of the RFT period was ≤32 mL/m^2^ (*p* = 0.013) (Table 4).

There were no other postoperative complications (cardiac or pulmonary) in either group, and no specific treatment was applied to any patient for AKI. The length of hospital stay was similar for the two groups. All patients were discharged on the fourth postoperative day following RALP.

## 4. Discussion

In the present study, the incidence of AKI was 9.1% in patients who received ≤3 h of RFT, and the incidence was 45.5% in patients who received >3 h of RFT. The results showed that the risk for AKI increased 12-fold when the duration of RFT was >3 h, the total fluid administered was ≤3.3 mL/kg/h, and the SVI at the end of the RFT period was ≤32 mL/m^2^. These results suggest that the prolongation of RFT and a decrease in the overall fluid balance can lead to AKI.

Although RFT has been widely studied in recent years, it remains unclear how much fluid and at which surgical stage it is administered are considered safe in terms of AKI, for specific surgeries such as RALP [9]. Postoperative AKI is not uncommon after major surgery. According to a recently published prospective international observational trial that included 10,000 patients, approximately one in five patients is diagnosed with postoperative AKI after major surgery [10]. Furthermore, the incidence of AKI is highest in patients undergoing urological and cardiovascular surgery [10]. RFT is an important risk factor for postoperative AKI. The risk for AKI may increase further in surgeries such as RALP, where the deep Trendelenburg position, pneumoperitoneum, and RFT are applied.

Fluid restriction in the RALP is a crucial requirement for reducing vesicourethral anastomosis complications, recovery of intestinal function, and controlling edema in the upper airway that may develop in the deep Trendelenburg position [2,11].

The most vulnerable period for patients in RALP is the console period due to the combination of pneumoperitoneum, deep Trendelenburg position, and fluid restriction that may affect the hemodynamics and renal blood flow. This triad needs to be assessed for the risk of AKI.

Pneumoperitoneum and the Trendelenburg position are potential risk factors for postoperative AKI, even without RFT. Animal and human studies suggest that both the deep Trendelenburg position and pneumoperitoneum can impair renal blood flow [12,13,14]. However, it is also known that laparoscopic surgical approaches reduce surgical stress and enhance recovery after surgery by reducing neuroendocrine and inflammatory responses [15]. In a retrospective analysis of 3692 patients, Essber et al. reported that, despite the potential mechanisms, the laparoscopic surgical approach with the Trendelenburg position did not reduce the postoperative estimated glomerular filtration rate, and it reduced the incidence of postoperative AKI compared to open surgery without the Trendelenburg position [16].

In the present study, we examined the hemodynamic effects of pneumoperitoneum and the Trendelenburg position, which may contribute to the development of AKI, using functional hemodynamic monitoring parameters. Cardiac index and macrohemodynamic parameters were similar in both groups at all time points. These findings emphasize the contribution of fluid therapy to the development of postoperative AKI. However, considering that postoperative AKI may have multifactorial causes, the possible contribution of the deep Trendelenburg position and pneumoperitoneum to the development of AKI in specific surgeries such as RALP should not be ignored.

In laparoscopic surgeries, urine flow decreases with gas insufflation and increases with desufflation [17]. However, in RALP, it is only possible to monitor urine output once urethral anastomosis is achieved. Thus, it cannot be considered an accurate monitoring parameter. Ahn et al. demonstrated that RALP does not cause postoperative renal dysfunction. Furthermore, even if RALP lasted >4 h with a pneumoperitoneum pressure of 15–20 mmHg, the creatinine and creatinine clearance values on the 30th postoperative day were similar to the preoperative values [18]. However, some studies have reported that AKI develops immediately after RALP with varying rates (13–46%) [6,19]. Furthermore, it remains unclear whether this changing risk for AKI is due to hemodynamic changes or it depends on the amount, duration, and timing of the RFT. Some studies have reported that despite the potential mechanisms of the Trendelenburg position and pneumoperitoneum, laparoscopic surgeries do not increase the risk of AKI without RFT [16]. The present study results highlight the importance of the effect of RFT duration in RALP on the risk of AKI. Additionally, we examined intraoperative hemodynamic findings that could predict this risk.

We divided our patients into two groups according to the RFT duration. The frequency of AKI was 9.1% in patients who received RFT for ≤3 h and it was 45.5% in patients who received RFT for >3 h. We examined the causes and indicators for this significant difference. During RALPs in our hospital, 1–1.5 mL/kg/h of crystalloid was administered until the end of the console period; after the anastomosis was achieved, fluids were administered liberally (approximately 8–10 mL/kg/h crystalloid).

The amount of fluid administered before and after the anastomosis was similar in both groups. However, when the total fluid administered was considered, it was significantly less in patients who received RFT for >3 h than in those who received RFT for ≤3 h. This indicates that although fluids were administered liberally after the anastomosis, the total fluid balance was inadequate if the console time was prolonged.

The Trendelenburg position is a nonphysiological position and may cause significant hemodynamic changes in combination with pneumoperitoneum [20]. Although the Trendelenburg position improves the hemodynamics by increasing venous return in hypovolemic patients, its long-term application with pneumoperitoneum may negatively impact the cardiovascular system performance [21]. The impact of the Trendelenburg position and pneumoperitoneum on CI is variable. While some studies have reported a significant increase in CI during RALP, others have reported a significant decrease in CI [22,23]. In our study, although the incidence of AKI was higher in patients who received >3 h of RFT than in those who received ≤3 h of RFT, the CI was similar between both groups at all time points. The CI is a relatively nonspecific measure of cardiac function. Thus, parameters such as CPO, CCE, Ea, and dp/dt, which reflect the cardiac power, efficiency, afterload, and contractility, respectively, should be considered in conjunction with the CI when assessing a patient’s cardiovascular status. In the present study, we monitored all these hemodynamic parameters, demonstrating the global cardiovascular performance of the patients. The CCE, an important parameter demonstrating the global cardiovascular performance over energy consumption, decreased gradually compared to the baseline in both groups. Additionally, it could not return to the baseline value at the end of the console period. These findings indicate that the triad of RFT, Trendelenburg position, and pneumoperitoneum increased the cardiac energy consumption and negatively affected the global cardiovascular performance.

We did not find any difference in the baseline hemodynamic parameter values before anesthesia between the two groups. Similarly, there was no significant difference in the hemodynamic parameter measurements after anesthesia induction and after 1 h in the Trendelenburg position. However, at the end of the console period (after RFT was discontinued), the HR and Ea were significantly higher in patients who received >3 h of RFT than in those who received ≤3 h of RFT. Furthermore, the SVI was significantly lower in patients who received >3 h of RFT than in those who received ≤3 h of RFT. The increase in HR while the CI remained unchanged can be considered a compensatory response to the decrease in SVI. The increase in Ea was also a compensatory mechanism in the vascular system for the decrease in SVI. Although the macrohemodynamic parameter values were similar, the higher HR and Ea values in patients who received >3 h of RFT may be considered signs of hypovolemia due to RFT prolongation. At the end of the console period, patients who received RFT for a longer period experienced a compensatory increase in the HR due to a fluid deficit.

We did not observe a significant increase in the risk of AKI when the total fluid administered was >3.3 mL/kg/h, even if the RFT was administered for >3 h. This indicates that the risk of AKI may be lower if the fluid deficit that develops due to RFT during the console period is replaced during the remaining anesthesia time. However, if the total fluid administered is not sufficient (<3.3 mL/kg/h) and the RFT duration is >3 h, the risk of AKI could increase by 10.8 times. Additionally, at the end of the console period, if the SVI was <32 mL/m^2^, the risk of AKI increased by 12 times.

This study had some limitations. We reported the results of RFT administration in RALP at our clinic. These results may vary with different RFT protocols. Thus, prospective randomized controlled studies comparing the different restrictive fluid protocols in RALP may be valuable. Additionally, we followed the patients in the first 48 h postoperatively. Thus, we do not have subsequent data of the patients who developed AKI. Finally, our patients were mostly healthy individuals (ASA I-II); the study results may vary in high-risk patients.

## 5. Conclusions

In conclusion, this prospective observational study demonstrated that prolonged RFT in RALP may increase the risk of AKI.

If RFT exceeds three hours during RALP, it may be beneficial to monitor patients for early signs of hypovolemia using dynamic hemodynamic parameters to identify patients at risk of developing AKI. Further studies are needed to clarify the impact of different fluid regimens and hemodynamic risk factors for developing postoperative AKI following RALP.

## Figures and Tables

**Table 1:** Comparison of the patients’ characteristics and preoperative laboratory test values between the two groups.

	RFT Period ≤ 3 h (*n* = 22)	RFT Period > 3 h (*n* = 22)	*p*
Age, years	66 (61–72)	66 (62–70)	0.860
BMI, kg m^−2^	27.1 ± 3.0	28.1 ± 4.5	0.393
ASA	2 (2–2)	2 (2–2)	0.780
Comorbidities, *n* (%)			
HT	15 (68.2)	16 (72.7)	1.000
DM	5 (22.7)	3 (13.6)	0.698
CAD	3 (13.6)	4 (18.2)	1.000
COPD	3 (13.6)	2 (9.1)	1.000
CVD	0 (0.0)	1 (4.5)	1.000
Preoperative laboratories			
Hb, g dL^−1^	14.0 ± 1.5	14.0 ± 1.3	0.958
Hct, %	41.6 ± 4.2	42.0 ± 3.7	0.732
Urea, mg dL^−1^	28 ± 11	31 ± 14	0.485
Creatinine, mg dL^−1^	0.91 ± 0.11	0.96 ± 0.13	0.174
Fasting, hour	11 (10–14)	10 (10–12)	0.169
Colonic cleansing, *n* (%)	21 (95.5)	21 (95.5)	1.000

RFT, restrictive fluid therapy; BMI, body mass index; ASA, American Society of Anesthesiologists; HT, hypertension; DM, diabetes mellitus; CAD, coronary artery disease; COPD, chronic obstructive pulmonary disease; CVD, cerebrovascular disease; Hb, hemoglobin; Hct, hematocrit; AKI, acute kidney injury.

**Table 2:** Comparisons of patients’ intraoperative parameters and outcomes of groups.

	RFT Period ≤ 3 h (*n* = 22)	RFT Period > 3 h (*n* = 22)	*p*
Baseline hemodynamic parameters			
HR, bpm	69 (62–76)	72 (66–79)	0.221
SAP, mmHg	154 ± 13	156 ± 17	0.282
DAP, mmHg	79 ± 8	77 ± 10	0.241
MAP, mmHg	100 ± 11	103 ± 11	0.189
SVI, ml/m^2^	48 ± 10	46 ± 13	0.610
CI, L/min/m^2^	3.0 (2.8–3.5)	3.0 (2.6–4.2)	0.851
Ea, L/min/m^2^	1.13 ± 0.27	1.09 ± 0.35	0.713
dp/dt, mmHg/msn	1.32 (1.22–1.56)	1.30 (1.11–1.80)	0.769
CPO, watt	1.40 (1.20–1.62)	1.56 (1.08–2.00)	0.690
CCE, unit	0.29 (0.10–0.44)	0.27 (0.07–0.51)	0.742
Surgery time, min	232 ± 42	293 ± 31	<0.001
Trendelenburg time, min	201 (169–210)	240 (223–256)	<0.001
Console time, min	150 (135–180)	230 (215–266)	<0.001
Intraoperative fluid administration,			
pre-anastomosis, mL kg^−1^ h^−1^	1.2 ± 0.3	1.0 ± 0.4	0.434
post-anastomosis, mL kg^−1^ h^−1^	9.7 (7.6–13.5)	10.7 (8.2–14.6)	0.091
Total, mL kg^−1^ h^−1^	4.3 ± 1.0	3.0 ± 0.9	<0.001
The usage of drugs during the intraoperative period, *n* (%)			
Ephedrine	18 (81.8)	17 (77.3)	1.000
Ephedrine (mg kg^−1^)	0.13 (0.06–0.38)	0.13 (0.05–0.27)	0.795
Norepinephrine	1 (4.5)	5 (22.7)	0.185
Norepinephrine (mcg kg^−1^)	0.0 (0.0–0.0)	0.0 (0.0–0.04)	0.103
Diuretics	21 (95.5)	21 (95.5)	1.000
Intraoperative cardiac events, n (%)			
Hypotension (MAP < 60 mmg)	8 (36.4)	8 (36.4)	1.000
Arrhythmia	0 (0.0)	1 (4.5)	1.000
Ischemic changes in ECG	0 (0.0)	0 (0.0)	NS
Postoperative laboratories test results at 24th hour			
Hb, g dL^−1^	12.8 (11.2–13.6)	11.9 (10.9–12.9)	0.323
Hct, %	36.6 (33.1–38.6)	35.3 (32.0–37.9)	0.459
Urea, mg dL^−1^	28 ± 10	37 ± 11	0.006
Creatinine, mg dL^−1^	1.0 ± 0.2	1.3 ± 0.3	0.007
Troponin-I, ng mL^−1^	0.006(0.004–0.06)	0.006 (0.004–0.011)	0.728
Total fluid intake at the end of the first 72 h after surgery, mL	8155 ± 1328	8038 ± 846	0.730
AKI, *n* (%)	2 (9.1)	10 (45.5)	0.016

RFT, restrictive fluid therapy; HR, heart rate; SAP, systolic arterial pressure_;_ DAP, diastolic arterial pressure; MAP, mean arterial blood pressure; Ea, arterial elastance; PPV, pulse pressure variation; SVV, stroke volume variation; CI, cardiac index; CPO, cardiac power output; CCE. cardiac cycle efficiency; SVI, stroke volume index; ECG, electrocardiogram; Hb, hemoglobin; Hct, hematocrit; AKI, acute kidney injury.

**Table 3:** Comparison of the hemodynamic parameters at different time points between the two groups.

Hemodynamic Parameters	RFT Period ≤ 3 h (*n* = 22)	RFT Period > 3 h (*n* = 22)	*p*
After anesthesia induction			
HR, min^−1^	61 ± 10	65 ± 12	0.271
SAP, mmHg	105 ± 18	104 ± 18	0.843
DAP, mmHg	60 ± 10	59 ± 8	0.815
MAP, mmHg	74 ± 14	73 ± 11	0.624
SVI, mL/m^2^	40 ± 9	36 ± 8	0.090
CI, L/min/m^2^	2.3 (2.2–2.6)	2.1 (2.0–2.4)	0.078
PPV, %	8 (7–12)	10 (8–16)	0.168
SVV, %	8 (7–9)	10 (6–13)	0.137
Ea, L/min/m^2^	0.95 (0.79–1.17)	1.03 (0.92–1.30)	0.366
dP/dt, mmHg/msn	0.76 (0.53–0.85)	0.72 (0.52–0.93)	0.869
CPO, watt	0.75 (0.67–0.94)	0.74 (0.60–0.80)	0.348
CCE, unit	0.06 ± 0.26	0.02 ± 0.28	0.580
After 1 h in the Td position			
HR, min^−1^	53 (49–59)	55 (52–62)	0.166
SAP, mmHg	96 ± 13	100 ± 15	0.271
DAP, mmHg	61 ± 8	66 ± 10	0.095
MAP, mmHg	73 ± 9	77 ± 12	0.175
SVI, mL/m^2^	45 ± 10	39 ± 8	0.074
CI, L/min/m^2^	2.4 ± 0.3	2.3 ± 0.3	0.320
PPV, %	11 ± 4	13 ± 5	0.156
SVV, %	9 ± 4	8 ± 3	0.512
Ea, L/min/m^2^	0.73 (0.68–0.98)	0.79 (0.76–1.01)	0.326
dP/dt, mmHg/msn	0.43 (0.28–0.58)	0.44 (0.31–0.50)	0.879
CPO, watt	0.77 ± 0.15	0.80 ± 0.16	0.451
CCE, unit	−0.21 ± 0.34	−0.30 ± 0.35	0.391
At the end of the console period			
HR, min^−1^	60 (51–67)	65 (59–71)	0.041
SAP, mmHg	92 ± 19	102 ± 20	0.105
DAP, mmHg	54 ± 12	60 ± 12	0.125
MAP, mmHg	67 ± 16	73 ± 14	0.134
SVI, ml/m^2^	38 ± 8	32 ± 8	0.016
CI, L/min/m^2^	2.2 ± 0.3	2.1 ± 0.4	0.232
PPV, %	8 (6–10)	9 (6–16)	0.494
SVV, %	11 (7–12)	8 (5–14)	0.160
Ea, L/min/m^2^	0.86 (0.70–0.99)	1.07 (0.94–1.23)	<0.001
dp/dt, mmHg/msn	0.46 (0.39–0.63)	0.48 (0.40–0.76)	0.372
CPO, watt	0.68 ± 0.20	0.69 ± 0.21	0.810
CCE, unit	−0.09 (−0.28, 0.22)	−0.27 (−0.40, 0.07)	0.197

RFT, restrictive fluid therapy; HR, heart rate; SAP, systolic arterial pressure; DAP, diastolic arterial pressure; MAP, mean arterial blood pressure; SVI, stroke volume index; CI, cardiac index; PPV, pulse pressure variation; SVV, stroke volume index; Ea, arterial elastance; CPO, cardiac power output; CCE, cardiac cycle efficiency; Td, Trendelenburg; h, hour.

**Table 4:** Relative risks for postoperative AKI.

	Relative Risk (CI 95%)	*p*
RFT ≤ 3 h plus	Ref	-
Total fluid > 3.3 mL/kg/h plus		
SVI at the end of the console period ≥ 32 mL/m^2^		
RFT period > 3 h plus	1.1 (0.1–22.7)	0.972
Total fluid > 3.3 mL/kg/h plus		
SVI at the end of the console period ≥ 32 mL/m^2^		
RFT period > 3 h plus	10.8 (1.4–82.6)	0.022
Total fluid ≤ 3.3 mL/kg/h plus		
SVI at the end of the console period ≥ 32 mL/m^2^		
RFT period > 3 h plus	12.0 (1.7–85.2)	0.013
Total fluid ≤ 3.3 mL/kg/h plus		
SVI at the end of the console period < 32 mL/m^2^		

AKI, acute kidney injury; CI, confidence interval; RFT, restrictive fluid therapy; SVI, stroke volume index.

## Data Availability

The datasets for the current study are available from the corresponding author upon reasonable request.

## References

[1] Ren S., Nathan S., Pavan N., Gu D., Sridhar A., Autorino R. (2022). Robot-Assisted Radical Prostatectomy: Advanced Surgical Techniques.

[2] Chiumello D., Fratti I., Coppola S. (2023). The Intraoperative Management of Robotic-Assisted Laparoscopic Prostatectomy. Curr. Opin. Anaesthesiol..

[3] Awad H., Walker C.M., Shaikh M., Dimitrova G.T., Abaza R., O’Hara J. (2012). Anesthetic Considerations for Robotic Prostatectomy: A Review of the Literature. J. Clin. Anesth..

[4] Seo D.Y., Cho H.J., Cho J.M., Kang J.Y., Yoo T.K. (2013). Experience with Robot-Assisted Laparoscopic Radical Prostatectomy at a Secondary Training Hospital: Operation Time, Treatment Outcomes, and Complications with the Accumulation of Experience. Korean J. Urol..

[5] Wiesenthal J.D., Fazio L.M., Perks A.E., Blew B.D.M., Mazer D., Hare G., Honey R.J.D., Pace K.T. (2011). Effect of Pneumoperitoneum on Renal Tissue Oxygenation and Blood Flow in a Rat Model. Urology.

[6] Sato H., Narita S., Saito M., Yamamoto R., Koizumi A., Nara T., Kanda S., Numakura K., Inoue T., Satoh S. (2020). Acute Kidney Injury and Its Impact on Renal Prognosis after Robot-Assisted Laparoscopic Radical Prostatectomy. Int. J. Med. Robot..

[7] Mori C., Iwasaki H., Sato I., Takahoko K., Inaba Y., Kawasaki Y., Tamaki G., Kakizaki H. (2023). Impact of Intraoperative Fluid Restriction on Renal Outcomes in Patients Undergoing Robotic-Assisted Laparoscopic Prostatectomy. J. Robot. Surg..

[8] Khwaja A. (2012). KDIGO Clinical Practice Guidelines for Acute Kidney Injury. Nephron.

[9] Wrzosek A., Jakowicka-Wordliczek J., Zajaczkowska R., Serednicki W.T., Jankowski M., Bala M.M., Swierz M.J., Polak M., Wordliczek J. (2019). Perioperative Restrictive versus Goal-Directed Fluid Therapy for Adults Undergoing Major Non-Cardiac Surgery. Cochrane Database Syst. Rev..

[10] Zarbock A., Weiss R., Albert F., Rutledge K., Kellum J.A., Bellomo R., Grigoryev E., Candela-Toha A.M., Demir Z.A., Legros V. (2023). Epidemiology of Surgery Associated Acute Kidney Injury (EPIS-AKI): A Prospective International Observational Multi-Center Clinical Study. Intensive Care Med..

[11] Gainsburg D.M. (2012). Anesthetic Concerns for Robotic-Assisted Laparoscopic Radical Prostatectomy. Minerva Anestesiol..

[12] Bishara B., Karram T., Khatib S., Ramadan R., Schwartz H., Hoffman A., Abassi Z. (2009). Impact of Pneumoperitoneum on Renal Perfusion and Excretory Function: Beneficial Effects of Nitroglycerine. Surg. Endosc..

[13] Abassi Z., Bishara B., Karram T., Khatib S., Winaver J., Hoffman A. (2008). Adverse Effects of Pneumoperitoneum on Renal Function: Involvement of the Endothelin and Nitric Oxide Systems. Am. J. Physiol. Regul. Integr. Comp. Physiol..

[14] Hirvonen E.A., Nuutinen L.S., Kauko M. (1995). Hemodynamic Changes due to Trendelenburg Positioning and Pneumoperitoneum during Laparoscopic Hysterectomy. Acta Anaesthesiol. Scand..

[15] Watt D.G., Horgan P.G., McMillan D.C. (2015). Routine Clinical Markers of the Magnitude of the Systemic Inflammatory Response after Elective Operation: A Systematic Review. Surgery.

[16] Essber H., Cohen B., Artis A.S., Leung S.M., Maheshwari K., Khan M.Z., Sessler D.I., Turan A., Ruetzler K. (2021). Renal Injury after Open versus Laparoscopic Non-Cardiac Surgery: A Retrospective Cohort Analysis. Braz. J. Anesthesiol..

[17] Nishio S., Takeda H., Yokoyama M. (1999). Changes in Urinary Output during Laparoscopic Adrenalectomy. BJU Int..

[18] Ahn J.H., Lim C.H., Chung H.I., Choi S.U., Youn S.Z., Lim H.J. (2011). Postoperative Renal Function in Patients Is Unaltered after Robotic-Assisted Radical Prostatectomy. Korean J. Anesthesiol..

[19] Naito A., Taguchi S., Suzuki M., Kawai T., Uchida K., Fujimura T., Fukuhara H., Kume H. (2020). Transient Acute Kidney Injury Observed Immediately after Robot-assisted Radical Prostatectomy but Not after Open Radical Prostatectomy. Mol. Clin. Oncol..

[20] Katayama S., Mori K., Pradere B., Yanagisawa T., Mostafaei H., Quhal F., Motlagh R.S., Laukhtina E., Grossmann N.C., Rajwa P. (2022). Influence of Steep Trendelenburg Position on Postoperative Complications: A Systematic Review and Meta-Analysis. J. Robot. Surg..

[21] Pawlik M.T., Prasser C., Zeman F., Harth M., Burger M., Denzinger S., Blecha S. (2020). Pronounced Haemodynamic Changes during and after Robotic-Assisted Laparoscopic Prostatectomy: A Prospective Observational Study. BMJ Open.

[22] Hofer C.K., Zalunardo M.P., Klaghofer R., Spahr T., Pasch T., Zollinger A. (2002). Changes in Intrathoracic Blood Volume Associated with Pneumoperitoneum and Positioning. Acta Anaesthesiol. Scand..

[23] Haas S., Haese A., Goetz A.E., Kubitz J.C. (2011). Haemodynamics and Cardiac Function during Robotic-Assisted Laparoscopic Prostatectomy in Steep Trendelenburg Position. Int. J. Med. Robot..

